# Magnetoreception: activated cryptochrome 1a concurs with magnetic orientation in birds

**DOI:** 10.1098/rsif.2013.0638

**Published:** 2013-11-06

**Authors:** Christine Nießner, Susanne Denzau, Katrin Stapput, Margaret Ahmad, Leo Peichl, Wolfgang Wiltschko, Roswitha Wiltschko

**Affiliations:** 1 FB Biowissenschaften, J.W. Goethe-Universität Frankfurt, Siesmayerstrasse 70, 60054 Frankfurt am Main, Germany; 2 Université Pierre et Marie Curie, Casier 156, 4 Place Jussieu, 75005 Paris, France; 3 Max Planck Institut für Hirnforschung, Max-von-Laue-Strasse 4, 60438 Frankfurt am Main, Germany

**Keywords:** magnetic compass, cryptochrome 1a, photoreceptor, flavin cycle, conformational change

## Abstract

The radical pair model proposes that the avian magnetic compass is based on radical pair processes in the eye, with cryptochrome, a flavoprotein, suggested as receptor molecule. Cryptochrome 1a (Cry1a) is localized at the discs of the outer segments of the UV/violet cones of European robins and chickens. Here, we show the activation characteristics of a bird cryptochrome *in vivo* under natural conditions. We exposed chickens for 30 min to different light regimes and analysed the amount of Cry1a labelled with an antiserum against an epitope at the C-terminus of this protein. The staining after exposure to sunlight and to darkness indicated that the antiserum labels only an illuminated, activated form of Cry1a. Exposure to narrow-bandwidth lights of various wavelengths revealed activated Cry1a at UV, blue and turquoise light. With green and yellow, the amount of activated Cry1a was reduced, and with red, as in the dark, no activated Cry1a was labelled. Activated Cry1a is thus found at all those wavelengths at which birds can orient using their magnetic inclination compass, supporting the role of Cry1a as receptor molecule. The observation that activated Cry1a and well-oriented behaviour occur at 565 nm green light, a wavelength not absorbed by the fully oxidized form of cryptochrome, suggests that a state other than the previously suggested Trp^•^/FAD^•^ radical pair formed during photoreduction is crucial for detecting magnetic directions.

## 1. Introduction

Birds use the geomagnetic field for directional orientation. The avian magnetic compass was first described for European robins, *Erithacus rubecula*, passerine migrants [1], and has since been demonstrated in numerous other bird species, also including non-migrants like domestic chickens, *Gallus gallus* [2] (for review, see [3]). This compass appears to be based on a radical pair mechanism [4,5]: absorption of a photon leads by electron transfer to the formation of a pair of radicals which occur in singlet and triplet states. The chemical balance between these two states depends on the alignment of the radical pair in the magnetic field and could thus convey information on magnetic directions.

The radical pair model [4] suggested the eye as the site of magnetoreception. Indeed, an involvement of the eyes and the visual system is supported by experimental evidence [6–8]. The first step, photon absorption, makes magnetoreception light dependent. Behavioural experiments showed that migratory passerines can orient in their migratory direction under short-wavelength light ranging from UV to 565 nm green; under longer wavelengths, they are disoriented (for review, see [9]). The same wavelength dependency is indicated in chickens [10].

As receptor molecule, Ritz *et al.* [4] had suggested cryptochromes, blue-light absorbing flavoproteins, because they were the only known photoreceptor molecules in animals that form radical pairs [11]. Cryptochromes have been found in chickens [12–14] and passerines (e.g. [15]; see [16] for review). In a recent immunohistochemical study [17], we could show that cryptochrome 1a (Cry1a) fulfils the basic requirements of the radical pair model as a magnetosensor [4]: Cry1a was found to be located at the discs in the outer segments of the violet cones in the retinae of chickens and of the UV cones in European robins [17]. Our antiserum was directed against the C-terminus of Cry1a and did not detect Cry1a in the inner segment where it is formed [17], raising the interesting possibility that the antiserum labelled exclusively the light-activated form of Cry1a. Cryptochromes undergo conformational changes at the C-terminal domains in the course of the flavin photocycle [18–20]. Possibly, the antiserum detected only conformational changes associated with Cry1a activation that concur with magnetic orientation. Therefore, we exposed chickens to different lighting protocols and then used the antiserum to look for differences in the amount of immunolabelled Cry1a in the retina.

## 2. Material and methods

For this study, we used 25 chickens between 18 and 22 days old, that is, of an age where chicks can be trained to magnetic directions, and thus have a functioning magnetic compass [2,21].

Before exposure, the chickens were kept in boxes without tops so that they had access to daylight. The exposure itself lasted about 30 min; it took place in outdoor sunlight, in total darkness or in light produced by light-emitting diodes (LEDs). This light was of narrow bandwidth (termed ‘monochromatic’ in our behavioural studies, e.g. [3,9,10]; for bandwidth, see table 1). The lights were of equal quantal flux, about 8 × 10^15^ quanta s^−1^ m^−2^, with the exception of UV light, which had a quantal flux of only 0.8 × 10^15^ quanta s^−1^ m^−2^. In one assay, the chickens were pre-exposed to total darkness for 30 min, and then exposed to light for only 5 min.

**Table 1.** Parameters of exposure to light and number of birds involved. Peak (qu1, qu3), peak wavelength and wavelengths with half the intensity; *N*, number of chickens; abbreviations used in the figures.

pre-treatment	light treatment	wavelength (nm) peak (qu1, qu3)	intensity (mW m^−2^)	duration (min)	*N*	abbreviation
daylight	darkness	—		30	3	D
daylight	sunlight	full spectrum		30	3	S
darkness^a^	UV	373 (368, 381)	0.3	5	2	D–UV
daylight	UV	373 (368, 381)	0.3	30	3	UV
daylight	blue	424 (403, 459)	2.4	30	2	B
daylight	turquoise	502 (486, 518)	2.1	30	3	T
daylight	green	565 (550, 583)	1.9	30	4	G
daylight	yellow	590 (571, 604)	1.8	30	2	Y
daylight	red	645 (625, 666)	1.7	30	3	R

^a^This pre-treatment in darkness lasted 30 min.

Immediately after the end of the exposure, the chickens were killed and their eyes were excised and opened under the same light condition as the exposure had been; only for the retinae of the chickens exposed to UV, D–UV and darkness was the preparation done under 645 nm red light. Fixation and further processing of the retinae followed the procedures described in detail by Nießner *et al*. [17], with the retinae fixed in the eyecups for the first hour in the light condition of the previous exposure (see the electronic supplementary material for details). Aldehyde fixation, as used here, terminates any ongoing reactions because the proteins are cross-linked.

Immunohistochemistry largely followed the procedure described for whole mounts by Nießner *et al*. [17]. We used the same antibodies, guinea pig Cry1a antiserum and goat antiserum sc-14363, to immunolabel Cry1a and violet opsin (SWS1), respectively. For a more detailed description of the procedures used and the respective controls, see the electronic supplementary material and [17]. All retinae were evaluated with a confocal laser scanning microscope (Zeiss LSM 510 META). We scrutinized the total surface of the retinae and found the labelling to be consistent across different retinae treated with the same light regime. Hence, we here illustrate only one representative example of each of the datasets.

## 3. Results and discussion

### 3.1. Labelled Cry1a under sunlight and in darkness

**Figure 1.**
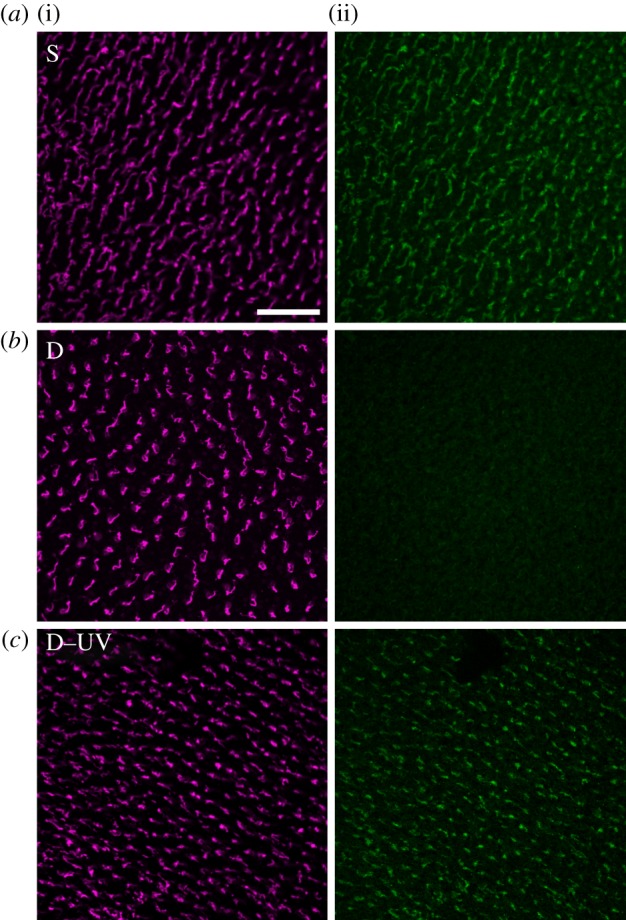
Double immunofluorescence labelling of chicken retinae for violet opsin marking the violet cones ((i) magenta fluorescence) and for Cry1a ((ii) green fluorescence). The two images in each row show the two labels in the same patch of retina. Treatment of the chickens: (*a*) pre-treatment in daylight, 30 min in sunlight (S); (*b*) pre-treatment in daylight, 30 min in total darkness (D); (*c*) 30 min pre-treatment in darkness, 5 min in 373 nm UV light (D–UV). The scale bar represents 50 µm (applies to all panels).

The signal intensity of the Cry1a antiserum labelling after exposure to sunlight, darkness and a brief light period following darkness is shown in figure 1. The signal obtained with the antiserum against the violet cone opsin is given for control. This antiserum always marked the violet cones; there was no indication of a treatment-dependent difference in the intensity of the opsin immunolabelling. By contrast, the amount of labelled Cry1a differed greatly: after exposure to sunlight, there was a high amount of marked Cry1a, whereas after exposure to darkness, no Cry1a was marked with our antiserum. Exposure to darkness, followed by a brief, 5 min exposure to light, again resulted in a considerable amount of Cry1a labelled by the antiserum.

The absence of Cry1a labelling after exposure to darkness raises the question whether darkness led to a degradation of Cry1a, or whether the epitope to which the antiserum binds was not accessible in the respective form. The quantity of labelled Cry1a in chickens that were kept 30 min in darkness, and then only briefly exposed to light clearly speaks in favour of the second possibility, as it seems highly unlikely that this amount of Cry1a can be produced and deposited in the outer segments of the violet cones within 5 min. However, if the epitope had been covered in darkness, it is easily conceivable that Cry1a is activated during this short interval and changes its conformation. The quantity of marked Cry1a in chickens that were only briefly exposed to light after being kept in darkness thus suggests that the different amounts of immunolabelled Cry1a were caused by a light-induced conformational change whereby the C-terminus becomes exposed to the surface of the protein [18], displaying the epitope for the antiserum to bind. This indicates that our antiserum against an epitope on the C-terminus does not label all Cry1a forms but only an activated form. A similar light-dependent conformational change in chicken cryptochrome 4 leads to recognition by a specific antibody which may hence be used as a conformational probe [14]. The same applies to our antiserum against the C-terminal of Cry1a.

### 3.2. Activated Cry1a under narrow-bandwidth lights of different wavelengths

**Figure 2.**
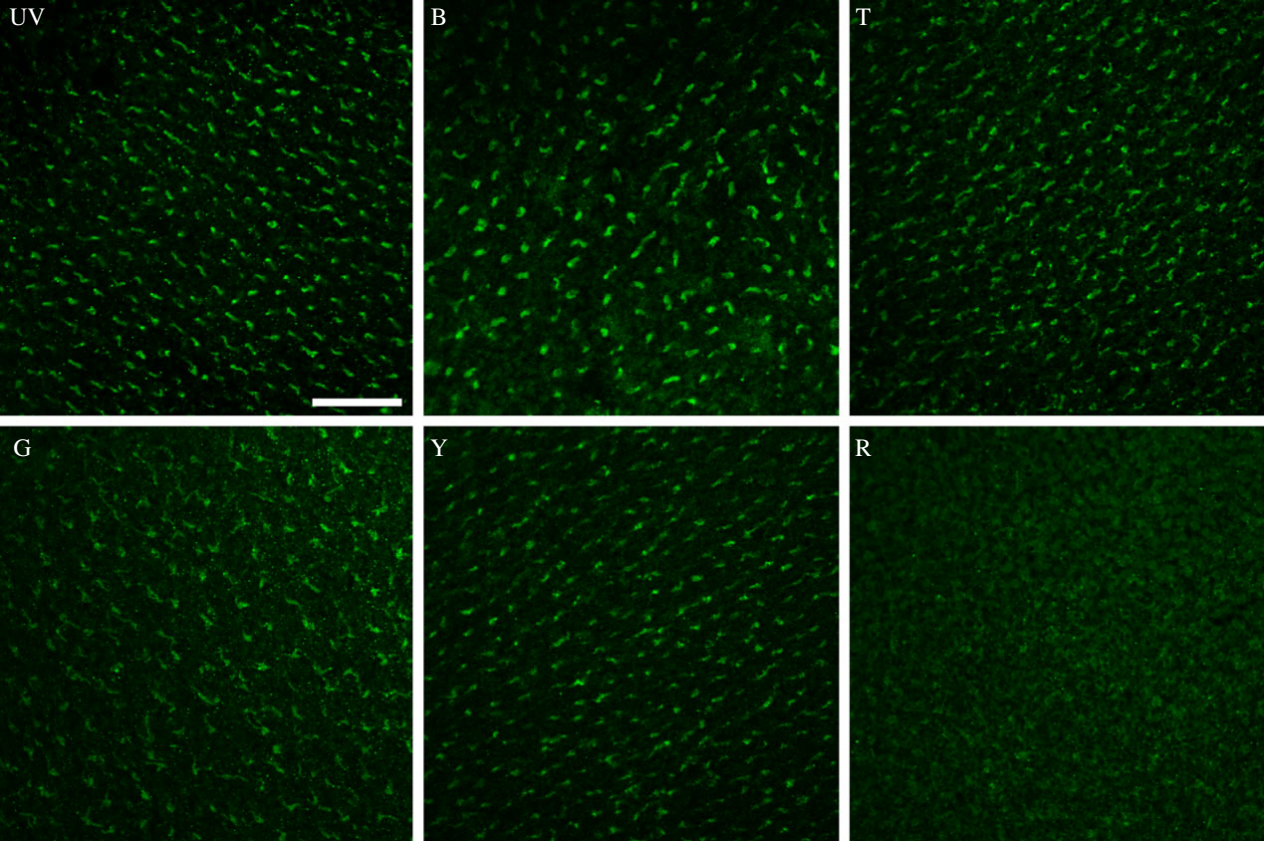
Amount of Cry1a immunolabelled with an antiserum against an epitope near the C-terminus of Cry1a after exposure to narrow-bandwidth lights of different wavelengths. UV, 373 nm ultraviolet; B, 424 nm blue; T, 502 nm turquoise; G, 565 nm green; Y, 590 nm yellow; R, 645 nm red (control labelling of these fields with the antiserum against violet opsin is shown in the electronic supplementary material, figure S1). The scale bar represents 50 µm (applies to all panels).

The amount of Cry1a that could be labelled after 30 min exposure to six light conditions is shown in figure 2; for the respective violet opsin controls, see the electronic supplementary material, figure S1. All of these wavelengths with the exception of red light resulted in labelling. The labelling intensity differed with wavelength: after exposure to 373 nm UV and 424 nm blue, there was a considerable amount of labelled Cry1a; with 502 nm turquoise, the amount of labelling appeared somewhat lower; and after exposure to 565 nm green and 590 nm yellow there seemed to be less activated Cry1a, indicated by the lower signal-to-noise ratio (higher background levels). Interestingly, the amount of activated Cry1a labelled under yellow appeared higher than that under green. After exposure to 645 nm red light, no activated Cry1a was visible. The labelling differences are more clearly seen at the microscope than in the printed micrographs in figure 2.

**Figure 3.**
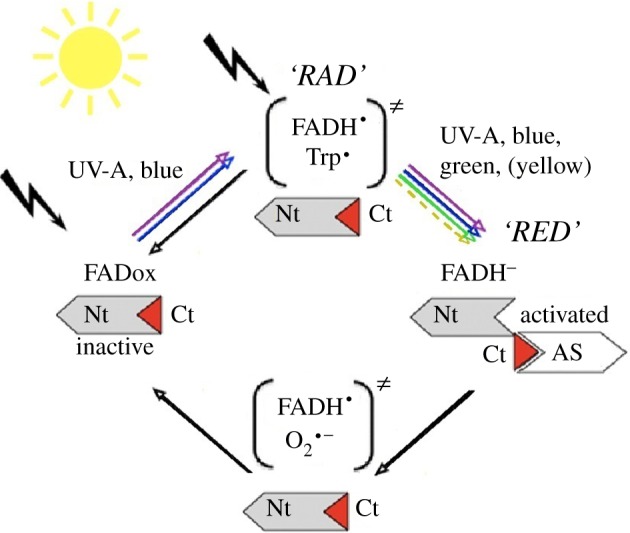
Flavin cycle of cryptochrome indicating where our antiserum (AS) might bind. Nt, nitrogen-terminus; Ct, carboxy-terminus of the protein, with the antiserum-binding epitope in red; in parentheses, radical pairs. Black arrows indicate light-independent reactions.

As expected, these findings reflect the light absorption characteristics of cryptochrome (figure 3). By analogy with other known cryptochromes, the following paradigm is assumed: in the dark, Cry1a is present with flavin in the fully oxidized form, FADox. Short-wavelength light from UV-A to about 500 nm reduces it to the radical redox state, the semiquinone intermediate RAD. This is a neutral radical FADH^•^ in isolated plant, algal and DASH-type cryptochromes (reviewed in [22]), but for animal-type cryptochromes from insects, an anionic semiquinone intermediate FAD^•−^ has been reported [23]. The neutral semiquinone of cryptochrome has light absorption characteristics ranging from UV-A through blue, green and yellow light up to around 600 nm; anionic semiquinone has a cut-off at shorter wavelengths, which, however, can extend into the green up to about 560 nm [23]. Oddly, the only bird cryptochrome analysed so far, gwCry1a from garden warblers, *Sylvia borin*, showed no absorption of green or yellow light (fig. 3 of [24]), which contrasts with the other spectroscopic measurements of cryptochrome [22,23] and our present data. Chicken Cry1a has not been analysed before. The semiquinone RAD can re-oxidize directly independently of light [25,26], whereas light absorption can further reduce RAD to the fully reduced form FADH^−^, RED. RED then re-oxidizes independently of light, completing the cycle (figure 3; see [26]).

The question now is which light-activated form of Cry1a does our antiserum label. Using the paradigm for cryptochrome activation by photoreduction as the basis for our model, the following mechanism can be inferred: as the first part of the study indicates, the fully oxidized form of Cry1a, which accumulates in the dark and is presumed to be inactive, is not labelled. Illumination by UV and blue light up to turquoise would, in principle, generate both the semiquinone and subsequently the fully reduced form, so that all redox forms of cryptochrome—FADox, RAD and RED—are present at the same time in a dynamic equilibrium determined by the light intensity. Because the chickens had been kept in daylight before treatment, we assume that a certain amount of the semiquinone RAD was present when the exposure to the specific light regimes began. Observing labelled Cry1a after 30 min exposure to green and yellow light, but not to red light indicates that green and yellow light can activate the receptor molecule even though these wavelengths are not absorbed by FADox. We cannot exclude that an unknown accessory pigment [27,28] absorbing in the green–yellow range is involved; yet no such pigment is known so far. The sole currently known mechanism consistent with our data is that cryptochrome in the bird retina absorbs light through both FADox and RAD, which drives photoreduction to the fully reduced form RED. It appears to be during the transition to the fully reduced form that the conformational change in the protein occurs, which is recognized by our antiserum (figure 3). This is different from most cryptochromes analysed so far, where the conformational change appears to occur when RAD is formed, which is assumed to be the signalling form [29–31]. However, recent data on an algal cryptochrome indicate that the redox transition from the neutral radical to fully reduced flavin can also initiate signalling in certain organisms [32]—this appears to be a parallel to what we assume for avian Cry1a when it is involved in magnetoreception.

It is interesting in this context that illumination with 590 nm yellow light leads to a somewhat stronger antiserum labelling than with 565 nm green light, which presumably should be better absorbed by the receptor molecule. Assuming that absorption by a limited pool of RAD indeed occurs, one explanation for this phenomenon would be that the labelled, fully reduced form RED has a relatively short half-life before it is re-oxidized independently of light. Green light, by promoting a faster redox transition from RAD to RED, would therefore deplete the available RAD pool more quickly and thereby accumulate less RED after a 30 min time period. The short half-life of RED may also be the reason for not finding labelled Cry1a after exposure to red light and in darkness: any RED present at the beginning would be re-oxidized within 30 min.

## 4. General discussion

Light absorption of cryptochromes during the various phases of the cycle has been analysed under laboratory conditions, with cryptochrome in solution [24,26,28,29] or in cell cultures [25,30,31]. Ours is the first study where the light exposure and the resulting activation of cryptochrome took place *in vivo* under natural conditions, with Cry1a inside the receptor cells in the retina of an intact eye at a body temperature of about 40°C. Hence, this immunohistochemical study reflects the responses of Cry1a in its natural context.

### 4.1. Wavelength dependency of Cry1a activation and magnetoreception in birds

The LEDs that we used here are the same as those we have used for behavioural studies with European robins [9]. Former experiments have indicated that robins and chickens have the same type of magnetic compass with the same wavelength dependency [10], and our previous immunohistochemical study [17] showed the same distribution of Cry1a in the retinae of both species. It is striking that with all wavelengths where robins are oriented by their inclination compass—from 373 nm UV to 565 nm green—we also find activated Cry1a, whereas we find no compass orientation in red light or in darkness. That means on the whole, the present immunohistochemical findings are in excellent agreement with the behavioural data, strongly supporting a role of Cry1a in magnetoreception.

The only exception is yellow light. Finding activated, labelled cryptochrome at this wavelength seems to suggest that the operational basis for the radical pair mechanism is provided, yet the robins are disoriented under this light condition [33]. The role of yellow light in sensing magnetic directions is unclear (see [9] for a detailed discussion). A similar phenomenon—long-wavelength light interfering with compass orientation—has also been observed in amphibians [34].

### 4.2. Implications for the radical pair mechanism of magnetoreception

Our findings lead to important novel implications concerning how cryptochromes can mediate magnetoreception in birds. It is generally accepted that in order to provide magnetic directional information, radical pairs must be formed [4,5]. Such radical pairs have been suggested to occur at least twice during the flavin photocycle [26] (figure 3). By absorption of UV, blue and also turquoise light, the oxidized form of flavin, FADox, generates the first radical pair FADH^•^/Trp^•^. In the case of plant cryptochromes, this reaction leads to a conformational change that forms the active, ‘lit’ state of cryptochrome (e.g. [18,30,31]). This is currently proposed for magnetoreception by cryptochrome [35–38]. Yet this radical pair is not consistent with our results, as we observe both Cry1a activation and magnetic orientation also in 565 nm green light, a wavelength that cannot generate the FADH^•^/Trp^•^ radical pair. It appears to be the illumination of the semiquinone RAD—the only form absorbing green light—that leads to obtaining magnetic information. If Cry1a is indeed the receptor molecule mediating magnetic directions in birds, then the FADH^•^/Trp^•^ radical pair may not in fact be the crucial one.

In a behavioural spectroscopy study subjecting robins to various radio frequency fields, Ritz *et al.* [39] found a strong resonance at the Larmor frequency of the electron. Subsequent calculations indicated that such a resonance occurs only in rather special radical pairs, namely when one of the partners is without magnetic nuclei. This seemed to point to the radical pair FADH^•^/O_2_^•−^ generated during re-oxidation, where one of the partners is oxygen. Hence, this radical pair was suggested to be the crucial one mediating magnetic directions [39]. Theoretical considerations, however, seem to speak against this possibility [40]. Yet, this radical pair would occur under green light as long as there was a sufficient quantity of the semiquinone available to be further reduced to RED, and thus provide the substrate for re-oxidation [26].

Our present data suggest that the activated form of avian Cry1a occurs when the flavin is fully reduced to RED [22], under light conditions that largely match the behavioural orientation of birds. The only currently known radical pair that may explain magnetic orientation consistent with these results is FADH^•^/O_2_^•−^ [26], formed during the re-oxidation of RED to FADox, restoring the receptor molecule to its inactive conformation. The reaction would be rendered potentially magnetically sensitive, because the ratio singlet/triplet, which depends on the alignment of the receptor molecule in the magnetic field, could affect the efficiency of re-oxidation. By this, it would modulate the amount of conformationally activated Cry1a, and consequently also the biological signal perceived by the bird. Such a type of mechanism, proposed by Ritz *et al*. [4], is in agreement with our present findings (but see also the models proposed by Stoneham *et al*. [38] and Hogben *et al*. [41]). Our present data thus provide additional support for the hypothesis of Cry1a being indeed the receptor molecule for magnetic directions.

## Notes

The study was performed according to the rules and regulations of Animal Welfare in Germany.
